# Sex hormones correlate with heart rate variability in healthy women and this correlation is conserved in women with well-controlled type 2 diabetes mellitus

**DOI:** 10.1371/journal.pone.0320982

**Published:** 2025-04-23

**Authors:** Adriana Robles-Cabrera, Claudia Lerma, Silvia Ruiz-Velasco Acosta, Iván Pérez-Díaz, Ruben Fossion

**Affiliations:** 1 Departamento de Fisiología, Facultad de Medicina, Universidad Nacional Autónoma de México, Mexico City, Mexico; 2 Centro de Ciencias de la Complejidad (C3), Universidad Nacional Autónoma de México, Mexico City, Mexico; 3 Departamento de Biología Molecular, Instituto Nacional de Cardiología Ignacio Chávez, Mexico City, Mexico; 4 Instituto de Investigaciones en Matemáticas Aplicadas y en Sistemas (IIMAS), Universidad Nacional Autónoma de México, Mexico City, Mexico; 5 Escuela de Medicina y Ciencias de Salud, Tecnológico de Monterrey, Mexico City, Mexico; 6 Departamento de Medicina, Instituto Nacional de Ciencias Médicas y Nutrición Salvador Zubirán, Mexico City, Mexico; 7 Instituto de Ciencias Nucleares, Universidad Nacional Autónoma de México, Mexico City, Mexico; University of Ljubljana, Medical faculty, SLOVENIA

## Abstract

The autonomic nervous system and the endocrine system regulate cardiovascular physiology, and their alterations, as occurs in type 2 diabetes mellitus, are related to the development of cardiovascular complications. Sex hormones are major regulators of both cardiovascular and nervous tissue, and during postmenopause, the lack in hormone production can increase the risk for cardiovascular and autonomic diseases, even more in metabolic impairment such as in T2DM However, the evidence regarding whether sex hormones are related to autonomic activity is inconclusive. The goal of this paper was to evaluate the correlation between sex hormones and cardiac autonomic activity, as assessed by heart rate variability (HRV), women with well-controlled type 2 diabetes (T2DM) and healthy women as the control group.

**Subjects and methods:**

In this study, four groups of women were designated according to their health status (control or T2DM) and fertility status (premenopausal or postmenopausal). Five serum sex hormones were measured (estradiol, progesterone, testosterone, LH and FSH), and time-domain and frequency-domain HRV indices were determined during three conditions: supine position, active standing, and rhythmic breathing. For the complete sample (n=118), bivariate Pearson correlations and linear multiple regressions were used to analyze the relationship between sex hormones, HRV indices, and other independent variables, such as glycemia and age. A p-value <0.05 was considered as significant.

**Results:**

There were no differences in sex hormones or HRV indices when comparing the healthy and T2DM groups. All bivariate Pearson correlations were significant between sex hormones and HRV indices; estradiol, progesterone, and testosterone have positive correlations; meanwhile, LH and FSH were negative in the time-domain (SDNN, RMSSD, pNN20) and frequency domain (PLF and PHF) indices. Regression models adjusted for mean heartbeat intervals confirmed an association between all sex hormones and HRV indices. Estradiol maintained significance in the regression models for specific HRV indices during supine and active standing conditions even after adjusting for age and glucose levels.

**Conclusions:**

All sex hormones correlate with HRV indices. Regression analysis confirms that this correlation is independent from the mean heartbeat interval. However, in regression models adjusted for age and glucose levels, only estradiol was found to be significant, and should be considered an important variable related to cardiovascular and autonomic balance in T2DM women and may provide crucial information to improve cardiovascular risk algorithms.

## 1. Introduction

Cardiovascular physiology is controlled by the endocrine system and the autonomic nervous system (ANS) [1] through the regulation of variables such as heart rate, cardiac muscle inotropism, vascular resistance, and blood volume [2]. In fertile women, sex hormones have periodic fluctuations that influence multiple regulatory systems including the endocrine, cardiovascular and nervous systems [3]. An inadequate level of hormones or nerve activity may dysregulate the cardiovascular system and increase susceptibility to disease [4,5]. During postmenopause (the physiological cessation of the ovarian sex hormones associated to aging), the risk of developing CVDs [6,7], and autonomic imbalance [8] increases rapidly, and even more in postmenopausal women with type 2 diabetes (T2DM) [9–11]. T2DM is a disease that affects multiple tissues including the autonomic branches that innervate the heart and this autonomic nerve damage is linked to deadly cardiac comorbidities such as silent ischemia, fatal arrythmias and sudden cardiac death [12–14] that could increase the mortality rate in T2DM postmenopausal women (Tawfik et al, 2015) were the lack of sex hormones also causes cardiovascular alterations. Glycemic control (known as HbA1c <7% or fasting blood glucose between 70–130 mg/dL [15]. PMID: 35891859; PMCID: PMC9304683.) is essential in reducing the risk of morbidity and mortality in T2DM, but only a relatively small proportion of the global population achieves good control, and this population is scarcely studied.

Cardiac autonomic activity can be assessed by studying the variations in heart rate. Heart rate variability (HRV) has been extensively investigated as a non-invasive, ambulatory, and low-cost biomarker of cardiac ANS activity. Specialized time-domain, frequency-domain and nonlinear statistical indices have been proposed to reflect specific sympathetic and parasympathetic aspects of HRV [16–18]. HRV can be evaluated in a basal resting state and during specific physiological challenges, such as active standing and rhythmic breathing [19–21].

Sex hormones are one of the most studied aspects of the endocrine system due to their pleiotropic tissular effects [22,23]. Despite the vast knowledge about the effect of sex hormones on cardiovascular physiology, there is limited information on a possible relationship between sex hormones and the activity of ANS measured via HRV indices. The evaluation of the presence of this relationship in health and disease, such as T2DM, is important to develop a biomarker combining this information which could be used to construct a cardiovascular risk calculator for T2DM women during postmenopause.

The association between sex hormones and HRV has been evaluated in healthy women, with inconclusive results [24–27]. This relationship has been poorly studied in diabetes, and only in type 1 diabetes [28] or gestational diabetes [29], but not in T2DM, where the effect of sex hormones [30] and HRV [31] has been separately studied only.

Our study hypothesized that the interaction between sex hormones, cardiovascular, and autonomic regulation can be approached as an integrative framework by exploring the correlations between serum sex hormones and HRV. The evidence in healthy premenopausal and postmenopausal women shows that HRV indices correlate with sex hormones. Estradiol, testosterone and progesterone had positive correlations; meanwhile, LH, FSH had negative correlations [24,26,32–34]. In these studies, the effect of aging was important because the production of sex hormones decreases with follicular senescence [35] and it has been studied that, in the autonomic nervous system, the sympathetic tone increases on the cardiovascular system, reducing the HRV [36]. Following this evidence, we hypothesized a positive correlation between HRV and estradiol, testosterone and progesterone, and a negative correlation between HRV and LH and FSH. The effect of T2DM on sex hormones has been less studied, and there are no previous reports in women with controlled T2DM. Therefore, no a priori hypothesis was proposed for the effect of T2DM on these correlations. The main goal of this study was to evaluate whether a relationship exists between serum sex hormones and HRV indices in women, considering their health status (healthy and well-controlled T2DM) and fertility stage (phases of the menstrual cycle in premenopause and postmenopause). In addition, all study variables were compared between groups to provide a comprehensive description of the study sample, identifying the main effects and interactions of healthy status, fertility stage and physiological maneuvers.

## 2. Subjects, materials and methods

### Study design

This study was observational, prospective, cross-sectional, and descriptive. This contribution presents a first attempt to describe the relationship between serum sex hormones and HRV in the context of T2DM. Women with T2DM were compared with healthy women. Only women with well-controlled T2DM were selected to avoid confounding factors such as polypharmacy, nephropathy, retinopathy, among others.

**Fig 1:**
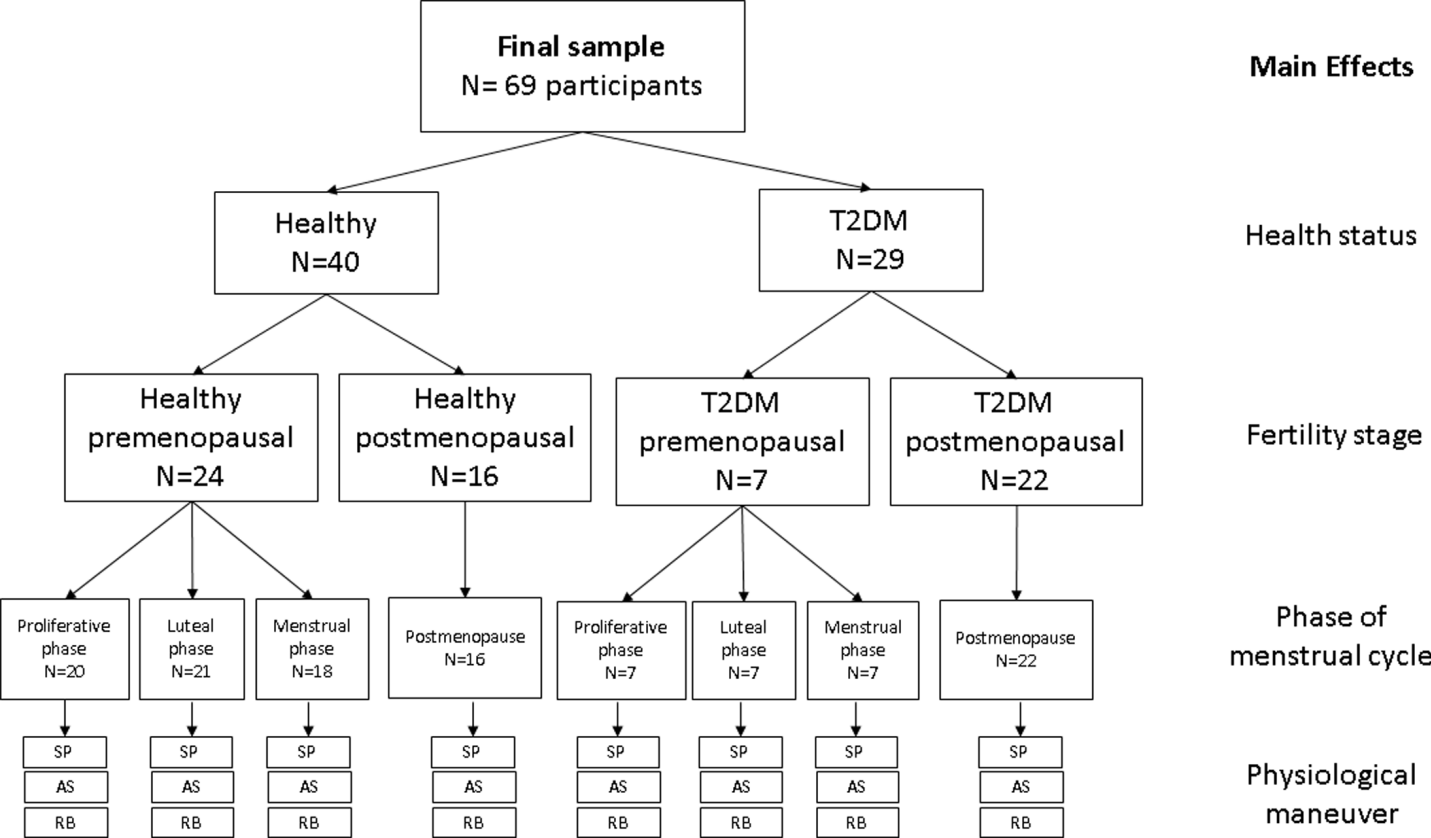
Flowchart of the study design and the steps and main effects performed. SP = supine position; AS = active standing; RB = Rhythmic breathing. *To perform the regression models, the mean of sex hormones and the mean of HRV indices were used.

The healthy and T2DM groups were each divided into two subgroups to separate premenopausal and postmenopausal women, representing different ages and hormonal productions. This resulted in four different groups: healthy premenopausal, T2DM premenopausal, healthy postmenopausal, and T2DM postmenopausal (Fig 1). Further analysis was conducted to explore the relationship between serum sex hormones and HRV indices, taking into consideration mean heart rate, age, and glucose level dependence.

Anthropometric features and clinical history were measured once for the entire sample. Sex hormone levels (17β-estradiol, progesterone, testosterone, luteinizing hormone and follicle-stimulating hormone), blood chemistry, and electrocardiogram (ECG) recordings were collected from premenopausal women in the three phases of the menstrual cycle (proliferative, luteal and menstrual) to represent normal hormonal production oscillations. Postmenopausal women were measured only once. ECG recordings were taken (to obtain HRV indices) from all participants during supine baseline and physiological challenges of active standing and rhythmic breathing, representing different conditions of autonomic cardiac regulation.

### Subjects

The study participants were recruited from December 2019 to July 2022 using a non-random convenience sampling method. Women with a normal ECG recording (defined as sinus rhythm, resting heart rate between 60–100 beats per minute (bpm), intervals, segments and waves within normal parameters and no extrasystoles exceeding 5%) and a body mass index (BMI) <30 kg/m^2^ were selected for all groups. Premenopausal women were aged between 35–50 years and had regular menstrual cycles, while postmenopausal women were aged between 40–60 years and had not experienced menstrual bleeding for at least 1 year. For the T2DM group, women with a confirmed diagnosis of type 2 diabetes according to the American Diabetes Association (glycated hemoglobin known as HbA1c >6.5%, or fasting plasma glucose >126 mg/dL, or oral glucose tolerance test >200 mg/dL over 2 hrs.), within the last 10 years, and HbA1c level of <7% (obtained from the clinical file) were included. The participants with T2DM were instructed to take their medication at the usual time the day before the study and to delay the dose on the study until data collection was completed.

The exclusion criteria comprised hormone treatments (such as contraceptives or hormone replacement therapy), pregnancy or lactation, diagnosed dysautonomia, intake of antihypertensive drugs or cardiac drugs, and other comorbidities or diseases (e.g., high blood pressure). T2DM patients treated with liraglutide or miglitol were also excluded.

We eliminate premenopausal women who showed any sign of menopause (such as irregular menstrual periods and hot flashes) or had abnormally high levels of follicle-stimulating hormone (FSH >22.51 mUI/mL) or luteinizing hormone (LH >103.03 mUI/mL) according to the phase of the menstrual cycle according to user guidelines for each hormone of Beckman Coulter.

### Materials

Information on anthropometric measurements, blood chemistry tests, sex hormone levels and ECG recordings were obtained for all participants.

Weight, BMI and metabolic rate were measured with an Omron HBF-514C scale, which is a validated scale for bioelectrical impedance analysis [37]. Blood pressure was measured with an Omron HEM-712C monitor, and height was measured with an InLab S50 stadiometer.

Blood samples were analyzed using spectrophotometry (filters 340–620 nm) with the Advia 1800 Clinical Chemistry System (Siemens, Munich, Germany). The following variables were measured: Glucose-hexocinase_3 (GLUH_3, Advia Chemistry, Siemens, Munich, Germany), HbA1c 3M (Advia 1800, Siemens, Munich, Germany), uric acid (Uricase/Peroxidase, Advia 1800, Siemens, Munich, Germany), creatinine (alkaline picrate, Advia 1800, Siemens, Munich, Germany), triglycerides (GPO-PAP method, Advia 1800, Siemens, Munich, Germany), total cholesterol, low-density lipoprotein cholesterol (LDL), and high-density lipoprotein cholesterol (HDL) (catalase method, Advia 1800, Siemens, Munich, Germany).

Sex hormone levels were measured using a paramagnetic particle chemiluminescence immunoassay: 17β-estradiol (estradiol-alkaline phosphatase conjugate, Beckman, Coulter, California, USA), progesterone (anti-progesterone antiserum in acetate buffer, Beckman, Coulter, California, USA), testosterone (anti-testosterone monoclonal antibody, Beckman, Coulter, California, USA), luteinizing hormone (LH) (anti-hLH antibody conjugate with alkaline phosphatase in saline buffer, Beckman, Coulter, California, USA) and follicle-stimulating hormone (FSH) (anti-hFSH-alkaline phosphatase conjugate, Beckman, Coulter, California, USA).

ECG recordings were obtained using the Zephyr Bioharness device (Zephyr Performance Systems, Medtronic, Annapolis, MD, USA).

### Methods

#### Ethical approval.

All procedures conducted in this study were approved by the Ethics and Research Committee of the Instituto Nacional de Ciencias Médicas y Nutrición Salvador Zubirán (INCMNSZ) in Mexico City, with the registration code CONBIOETICA-09-CEI-011–20160627, reference 3102. The latest revision of the Declaration of Helsinki (modified in 2013) and the Regulation of the General Law of Health in Research of México (modified in 2014) were considered. This project was low risk (article 17 section II) under the latter regulation due to venipuncture for blood sampling. All participants were extensively informed about the benefits, risks, and voluntary participation in the study, and all provide written informed consent.

The complete protocol was carried out between 7:00 and 9:00 a.m. at a room temperature ranging from 20 to 25 ^◦^C. Participants were required to fast and refrain from exercising, as well as consuming coffee, alcohol, or tobacco on the day before the study. Subjects in the diabetic group were instructed to take their normoglycemic medication after the study. In the T2DM group, 22 of 29 patients (76%) used metformin only as a normoglycemic treatment, 2 patients (7%) used metformin + Dipeptidyl peptidase-4 inhibitor, 2 patients (7%) used metformin + sodium glucose transporter inhibitor, 3 patients (10%) were controlled only with diet and exercise, and none of the patients were treated with insulin.

For each participant, a brief clinical history was established, with the focus on gynecological history, diseases, medications, T2DM duration, drug intake, and comorbidities. In premenopausal women, separate measures were taken during the proliferative, luteal and menstrual phases. To estimate the menstrual cycle, the first day of the last period was asked for in the clinical history, and days of the mid-follicular (day 10–12 of the cycle), mid-luteal (day 20–22) and menstrual phases (first and second day of bleeding) were calculated.

After the clinical questionnaire, anthropometric measurements were obtained, and the ECG recording protocol and the maneuvers were explained to the participants. A Zephyr Bioharness band was placed on the chest of the participants. The quality of the ECG signal and the stationarity of physiological variables such as heart rate, breathing rate, and movement were visually verified in real-time using the Zephyr Sensors application of the IoTool Platform for Android cellphones (SenLab, Slovenia, Balkans). To initiate the recording protocol, the participants were asked to lie down, remain silent and close their eyes.

The HRV protocol was conducted following specific considerations [20,38] to evaluate a baseline condition (supine position), followed by two physiological challenges (active standing and rhythmic breathing) to assess the effect of the activation of specific branches of the ANS (sympathetic and parasympathetic, respectively). The ECG was recorded continuously, with 10 minutes in each condition, where the first 5 minutes allowed heart rate to adapt to the new condition, and the HRV indices were calculated for the last 5 minutes of each condition.

After the ECG protocol, two blood samples were taken to analyze basic routine chemistry (glucose, HDL-cholesterol, LDL-cholesterol, total cholesterol, triglycerides, uric acid, creatinine and urea nitrogen) and sex hormone levels (17β-estradiol, progesterone, testosterone, LH and FSH).

The authors did not have access to any information that could identify individual participants during or after data collection, except for the first author who recruited the participants. Each participant was assigned an identification code to ensure anonymity in the database, which was analyzed by the co-authors. All measurements were performed by the same researcher to reduce observer bias.

#### ECG processing and HRV analysis.

The ECG was recorded at a sampling frequency of 250 samples per second using the Zephyr Bioharness device and ECG recordings were downloaded to a computer (Medtronics, Annapolis, MD, USA). A previously validated computer program was utilized to automatically identify each QRS complex [39], which was followed by visual inspection to identify any miss-detected beats that were then corrected manually. The resulting intervals measured between QRS and the next QRS, also known as RR intervals, were calculated and an adaptive filter was used to identify and correct RR intervals originating from ectopic sources, to obtain a final RR interval time series consisting of only sinus rhythm heartbeats.

The estimation of HRV indices was performed with a program developed in Matlab R2020b, following the recommendations for HRV analysis described in Malik, et al 1996 [16] and Robles-Cabrera et al., 2021 [20]. Time-domain HRV indices included: MeanNN (the mean of all RR intervals), SDNN (the standard deviation of all RR intervals), RMSSD (the square root of the mean of the squared differences between adjacent R-R intervals), and pNN20 (the percentage of successive RR intervals with differences larger than 20 ms). Frequency-domain HRV indices included: LF (the power in the low frequency band, 0.04 to 0.15 Hz), HF (the power in the high frequency band, 0.15 to 0.4 Hz), and the LF/HF ratio. LF and HF were calculated in absolute (ms^2^) and in normalized units (n.u.) [16]. The estimation of the power spectral density to obtain these frequency-domain HRV indices required the following steps: (i) detrending by linear interpolation, (ii) resampling at three samples per second, (iii) applying a Hanning window (300 data points, 50% overlap), and (iv) calculating the power spectrum density by the Welch periodogram method.

### Statistical analysis

The sample size for the study was determined based on the focus of the study, which was to examine the relationship between sex hormones and HRV indices. The calculation of the sample was based on a published study [24], which investigated the correlations between sex hormones and HRV indices in women and reported significant correlations between estradiol and HRV indices. Using the information from that study, here a minimum Pearson correlation of 0.40, an alpha error = 0.05 (two-tailed), and a beta error of 0.2 (statistical power of 0.8) were considered. As a result, a minimum of 47 participants were needed to estimate the relationship between sex hormones and HRV indices for the complete sample. A total of 69 participants were recruited for the study.

**Fig 2:**
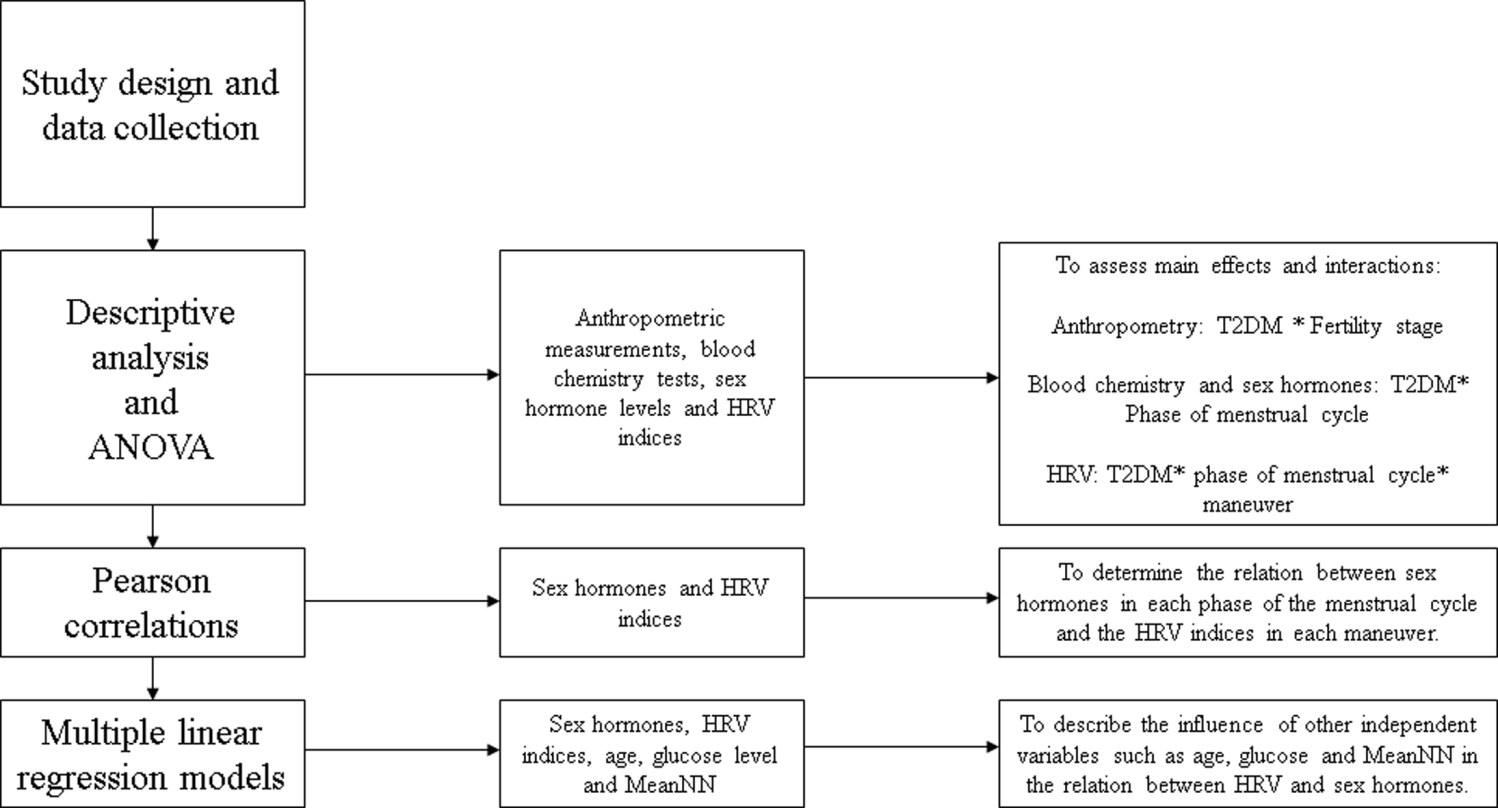
Flowchart of the statistical analyses performed step by step. HRV = heart rate variability, T2DM = type 2 diabetes mellitus.

A flowchart of the statistical analyses is presented in Fig 2. All analyses were performed with the software Statistical Package for Social Sciences (SPSS) version 25.0 (IBM, Armonk, NY, USA). P-value <0.05 was considered statistically significant. Normal distribution of the data was verified using t-Smirnov test and the Shapiro-Wilk test. Variables that did not follow a normal distribution were log-transformed. All data are shown as mean (standard deviation, SD). Comparisons between groups were performed using analysis of variance (ANOVA), the p-value of all post-hoc comparisons was adjusted using the Bonferroni method. For comparisons of HRV indices with three conditions (supine position, active standing, or rhythmic breathing), ANOVA for repeated measures was used.

The main goal of this work was to evaluate the relationship between sex hormones and HRV indices using both bivariate Pearson correlation (r) and multivariate regression analysis. The bivariate Pearson correlation analyses included samples of sex hormones and HRV across the three phases of the menstrual cycle and postmenopause to increase the dynamic range in the fluctuations of sex hormones (n=118). It is important to mention that all the interactions were non-significant and were therefore excluded from the regression models.

In the correlation analysis, statistical significance was determined using a p-value threshold of 0.05. The null hypothesis for the Pearson correlation test states that the correlation coefficient is equal to zero (i.e., no association between the variables). Correlation was considered to be significant (p<0.05) or very significant (p<0.001), and correlation magnitude was considered to be negligible (r <0.09), weak (0.10 < r < 0.39), moderate (0.40 < r < 0.69), strong (0.70 < r < 0.89) and very strong (0.90 < r < 1.0).

The regression model analysis included averaged samples of sex hormones and HRV indices from the three phases of the menstrual cycle (in the premenopausal group) and the sample measured in the postmenopausal group (n=69). This approach equalizes the sampling from the premenopausal and postmenopausal groups in the regression models. Considering that the three physiological conditions (supine position, active standing, and rhythmic breathing) represent different autonomic states, the correlation and regression analyses were performed separately for each condition.

Only variables with significant bivariate correlations were considered for the multiple regression models. Collinear variables with a variance inflation factor <3 were excluded from the multiple regression models. To account for possible correlations between hormones, age and glucose levels, two separate models were calculated to predict each HRV index as the dependent variable. The models included the following independent variables: (i) MeanNN and sex hormone levels; and (ii) MeanNN, sex hormones, age, and glucose level.

If a participant had missing data for a specific variable, they were excluded from the analyses involving that variable but were still included in the analyses of all other variables.

## 3. Results

### Comparisons between groups

The study sample consisted of 69 women divided into 4 groups: healthy premenopausal women (n=24), T2DM premenopausal women (n=7), healthy postmenopausal women (n=16), and T2DM postmenopausal women (n=22). Blood chemistry, sex hormone levels, and ECG protocols were measured separately for each of the three menstrual phases for the participants of the premenopausal groups and once for the participants of the postmenopausal groups, resulting in a total of n=118 measurements in the entire sample. In the women with T2DM, the mean time since the diagnosis of T2DM was 3.3 years (SD 2.6 years), and the mean level of HbA1c was 6.18 (SD 0.47) confirming glycemic control.

Table 1 shows results for anthropometry, the comparation between groups was developed with ANOVA and Bonferroni correction. There is an effect of the T2DM condition, but only in the premenopausal stage, where women with T2DM have a larger weight, higher BMI, higher overall fat percentage, higher visceral fat, and higher metabolic rate. There is an effect of menopause and aging: visceral fat is higher, and height is lower during postmenopause with respect to premenopause, both for healthy women and for T2DM women; BMI is higher in postmenopause for healthy women.

**Table 1:** Anthropometric results comparing groups. The results are presented as Mean (SD). n=69 participants. ANOVA with Bonferroni correction.

Variables	Healthy		T2DM	
	Premenopausal healthy n=24	Postmenopausal healthy n=16	Premenopausal T2DM n=7	Postmenopausal T2DM n=22
Age (years)	39.8 (6.4)	53.4 (3.0)^b^	40.4 (4.3)	54.9 (4.2)^b^
SBP (mmHg)	119.9 (12.5)	124.5 (18.6)	116.3 (10.5)	123.0 (16.5)
DBP (mmHg)	76.6 (8.9)	78.6 (8.6)	75.0 (7.6)	75.0 (10.3)
Height (m)	1.62 (0.06)	1.57 (0.07)^b^	1.62 (0.06)	1.55 (0.06)^b^
Weight (kg)	60.9 (7.5)	63.1 (8.4)	70.4 (4.6)^a^	63.5 (9.4)
BMI (kg/m^2^)	23.3 (2.0)	25.5 (3.1)^b^	27.0 (3.5)^a^	26.4 (2.8)
Heart rate (bpm)	62.1 (8.1)	65.1 (6.6)	66.3 (6.3)	68.2 (8.0)
Fat (%)	35.3 (5.1)	37.9 (4.7)	40.1 (6.3)^a^	41.2 (3.9)
Muscle (%)	26.1 (2.7)	25.3 (2.2)	25.5 (2.6)	24.3 (1.4)
Metabolic rate (kcal/day)	1300.1 (93.0)	1299.5 (93.2)	1403.5 (44.0)^a^	1331.6 (144.3)
Visceral fat (a.u.)	5.5 (1.3)	7.5 (1.7)^b^	7.0 (1.9)^a^	8.6 (1.6)^b^

SBP = systolic blood pressure; DBP = diastolic blood pressure; BMI = body mass index

^a^p < 0.05 T2DM compared to healthy group with same fertility stage (premenopause or postmenopause).

^b^p < 0.05 Postmenopausal compared to premenopausal group with same healthy status (healthy or T2DM)

Table 2 shows the results for blood chemistry with few differences between groups (ANOVA, Bonferroni correction). There is an effect of health status, but only for glucose levels, which are consistently higher for women with T2DM during postmenopause and for all phases of premenopause. There is an effect of menopause and aging, but only for healthy women, where LDL-cholesterol was lower in the menstrual phase and triglycerides were lower in the luteal and menstrual phases compared to postmenopause.

**Table 2:** Blood chemistry measurements. The results are presented as Mean (SD) or Median (percentile 25- percentile 75). ANOVA with Bonferroni correction.

Variables	Healthy group				T2DM group			
	Premenopause			Postmenopause n=16	Premenopause			Postmenopause n=22
	Proliferative n=20	Luteal n=21	Menstrual n=18		Proliferative n=7	Luteal n=7	Menstrual n=7	
Log HDL (mg/dL)	1.65 (0.19)	1.67 (0.16)	1.63 (0.19)	1.61 (0.13)	1.66 (0.07)	1.64 (0.08)	1.54 (0.13)	1.68 (0.09)
HDL (mg/dL)	41.30 (33.40-57.40)	46.70 (38.35-59.60)	43.90 (34.60-52)	41.10 (30.60-54.30)	42.90 (39.90-55.60)	42.10 (38.50-47)	38.50 (30.20-43.30)	49.65 (42.70-54.50)
LDL (mg/dL)	90.05 (34.56)	93.06 (37.59)	84.95 (29.97)^b^	116.13 (29.15)	91.94 (22.90)	105.53 (26.65)	91.43 (29.81)	105.51 (32.79)
Total cholesterol (mg/dL)	150.36 (45.72)	154.05 (44.80)	140.25 (40.99)	167.75 (28.99)	148.57 (17.95)	154.71 (29.62)	134.86 (33.13)	168.09 (37.01)
Log Triglycerides (mg/dL)	1.91 (0.18)	1.88 (0.15)^b^	1.87 (0.15)^b^	2.05 (0.18)	2.01 (0.22)	1.95 (0.17)	1.99 (0.19)	2.12 (0.17)
Triglycerides (mg/dL)	78(60-97)	75.50(55.50-87)	79(62-88)	108(77-156)	108(82-152)	76(65-144)	104(70-125)	119.50(107-167)
Uric acid (mg/dL)	3.62 (0.85)	3.86 (0.91)	3.69 (1.20)	3.81 (0.79)	4.04 (1.19)	4.25 (1.20)	3.50 (1.01)	4.50 (1.38)
Creatinine (mg/dL)	0.60 (0.20)	0.67 (0.25)	0.58 (0.19)	0.61 (0.16)	0.70 (0.14)	0.67 (0.12)	0.57 (0.20)	0.63 (0.15)
Glucose (mg/dL)	67.95 (16.38)	72.73 (15.79)	68.25 (19.00)	80.31 (22.01)	102.44 (18.86)^a^	101.17 (29.58)^a^	100.14 (27.61)^a^	100.64 (18.81)^a^
BUN (mg/dL)	10.09 (2.93)	11.14 (3.94)	10.40 (3.68)	12.25 (3.44)	11.96 (4.34)	14.00 (3.41)	11.71 (4.03)	14.10 (4.66)

T2DM = Type 2 diabetes mellitus; HDL = high density cholesterol; LDL = low density cholesterol; BUN = blood urea nitrogen

^a^p < 0.05, women with T2DM compared to healthy women (same cycle phase)

^b^p < 0.05, premenopausal menstrual cycle phases compared to postmenopause in same group (healthy or T2DM)

**Fig 3:**
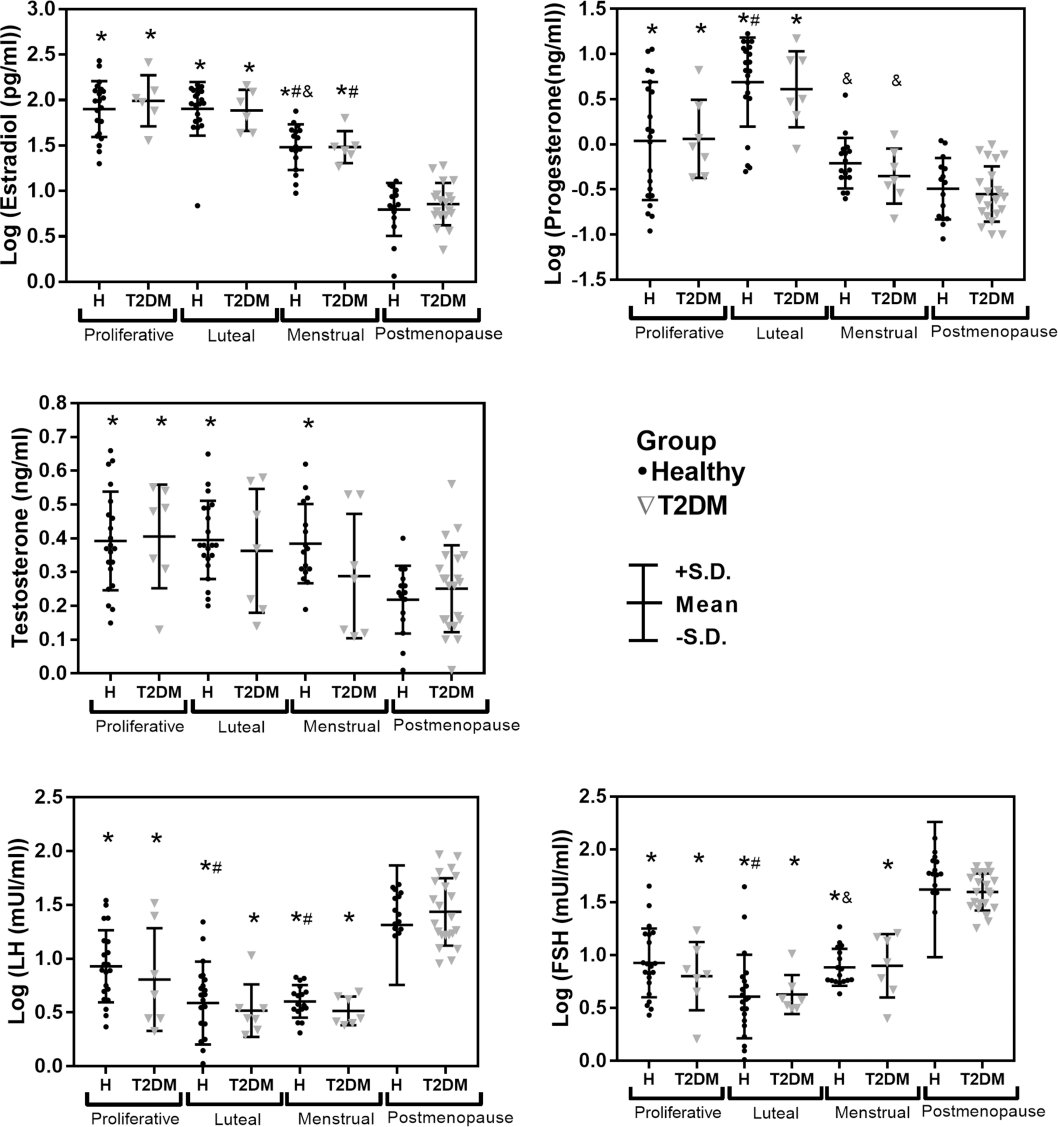
Comparison of sex hormone levels (Mean ± SD) between the different participant groups. For all sex hormones, the main effect of the menstrual cycle phase was significant (p < 0.001) while the main effect of T2DM was non-significant (p > 0.1) and the interaction term (menstrual cycle phase * T2DM) was non-significant (p >0.1). ANOVA with Bonferroni correction. * p < 0.05 vs postmenopause (in the same health status: healthy or T2DM). ^#^ p < 0.05 vs proliferative phase (in the same health status: healthy or T2DM). ^&^ p < 0.05 vs luteal phase (in the same health status: healthy or T2DM).

Fig 3 displays results for sex hormone levels, and no differences were found between healthy women and women with T2DM in the ANOVA analysis with Bonferroni correction. The effect of menopause and aging is reflected in lower levels of estradiol, progesterone, and testosterone, as well as higher levels of FSH and LH in postmenopausal women when compared to premenopausal women across most phases of the menstrual cycle. Within the menstrual cycle phases, sex hormone levels followed the expected physiological trends, with lower levels of estradiol and progesterone observed during the menstrual phase compared to the proliferative and luteal phases. All sex hormone serum levels were within the expected range for each cycle phase and postmenopause, except for three participants with low levels of progesterone in luteal phase. However, we found consistency in the menstrual and proliferative phases, indicating normal menstrual cycles that fulfilled the inclusion criteria, and these participants exhibited no symptoms or signs that would exclude them.

**Fig 4:**
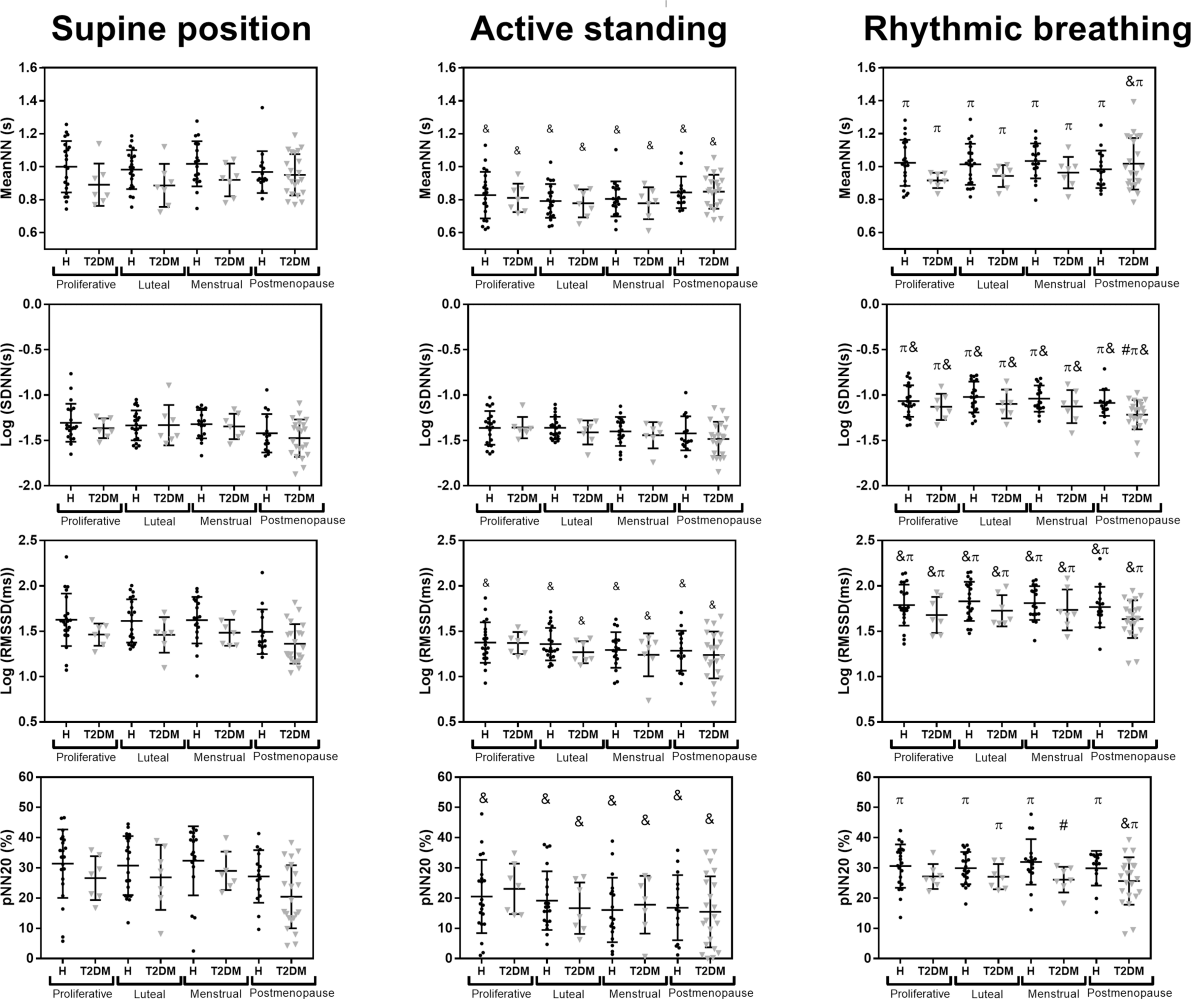
Comparison of time-domain HRV indices between healthy women and women with T2DM in postmenopause and each phase of the menstrual cycle. The main effect of the menstrual cycle phase, and T2DM, and the interaction term (menstrual cycle phase * T2DM) were non-significant for all HRV indices (p>0.1). The main effect of T2DM was significant for MeanNN and RMSSD (p<0.01), but non-significant for SDNN and pNN20. The main effect of the maneuver was significant for all indices (p<0.05), and the interaction terms (maneuver * T2DM; maneuver * menstrual cycle phase; maneuver * T2DM * menstrual cycle phase) were non-significant (p>0.5). ANOVA with Bonferroni correction. ^#^ p<0.05 vs. healthy (same menstrual phase and maneuver). ^&^ p<0.05 vs. supine position (same health status and menstrual phase). ^π^ p<0.05 vs. active standing (same health status and menstrual phase).

Fig 4 shows results for time-domain HRV indices. There are almost no differences between healthy women and women with T2DM for any of the phases of menstrual cycle and any of the physiological conditions. However, there are consistent significant differences between physiological conditions. Compared to the supine position, during active standing, there is a significant decrease in MeanNN, RMSSD, and pNN20 for all groups. There is a significant increase in SDNN and RMSSD for all groups during rhythmic breathing compared to the supine position. Compared to active standing, rhythmic breathing increases MeanNN, SDNN, and RMSSD for all groups, and pNN20 increases for all phases of the menstrual cycle in healthy women and the luteal phase and postmenopause in women with T2DM.

**Fig 5:**
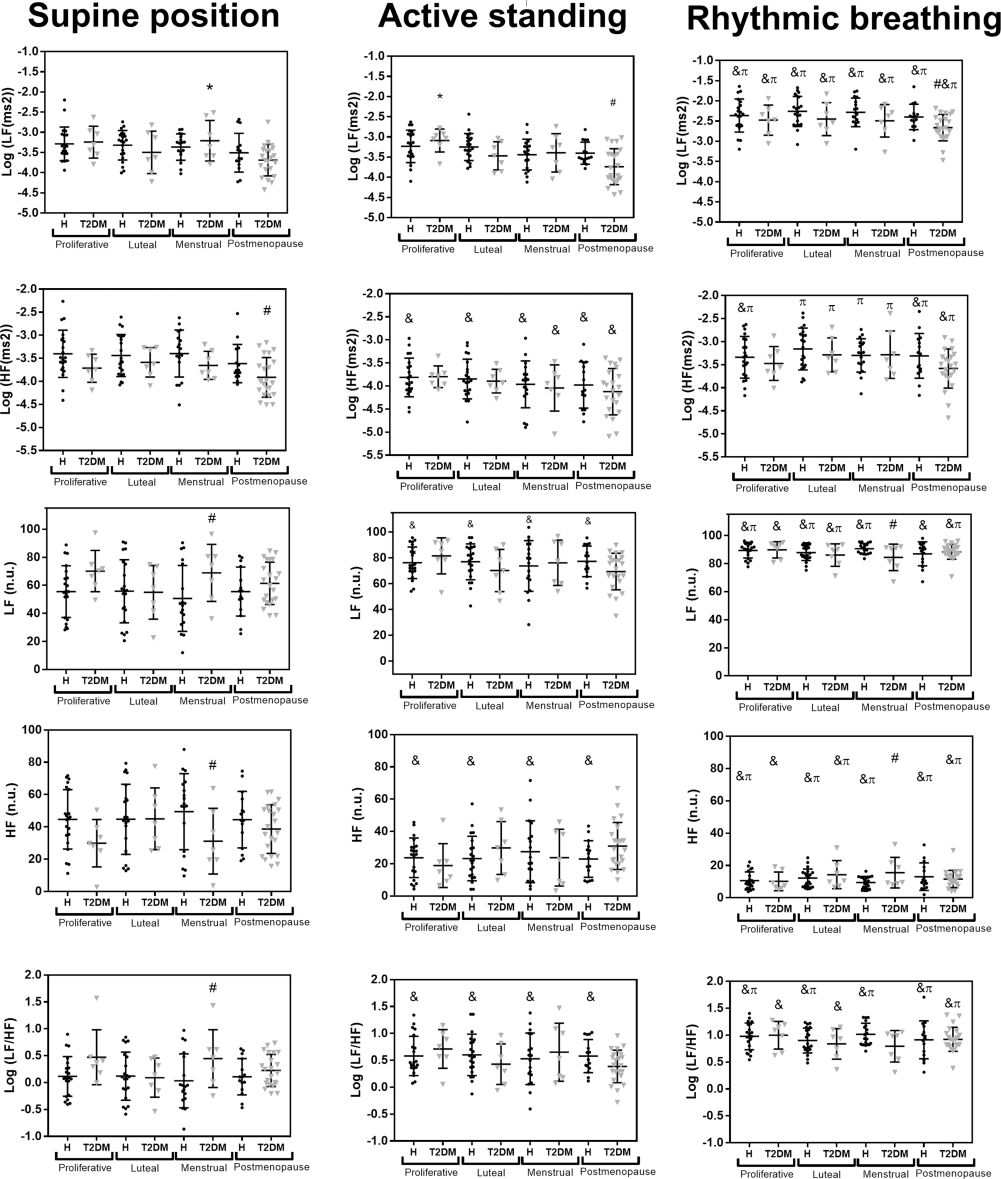
Comparison of frequency-domain HRV indices between healthy women and women with T2DM during postmenopause and each phase of menstrual cycle. The main effect of the menstrual cycle phase, and T2DM, and the interaction term (menstrual cycle phase * T2DM) were non-significant for all HRV indices (p > 0.1). The main effect of the maneuver was significant for all HRV indices (p<0.05), but the interaction terms (maneuver * T2DM; maneuver * menstrual cycle phase; maneuver * T2DM * menstrual cycle phase) were not significant for any of the HRV indices (p>0.5). ANOVA with Bonferroni correction. * p<0.05 vs. postmenopause for the same health status (healthy or T2DM) and maneuver (supine position, active standing or controlled breathing). ^#^ p<0.05 vs. healthy (same menstrual phase and maneuver). ^&^ p<0.05 vs. supine position (same health status and menstrual phase). ^π^ p<0.05 vs. active standing (same health status and menstrual phase).

Fig 5 displays the results for frequency-domain HRV indices. There are almost no differences between healthy women and women with T2DM for any of the phases of menstrual cycle or any of the physiological conditions. However, there are consistent significant differences between physiological conditions. Compared to the supine position, active standing decreases HF (ms^2^) in all healthy women and women with T2DM (only during the menstrual phase and postmenopause). Active standing also increases LF (n.u.), and LF/HF, but decreases HF (n.u.) in all healthy women. Rhythmic breathing increases LF (ms^2^), HF (nu), and LF/HF, but decreases HF (n.u.) for all groups and menstrual cycle phases compared to the supine position. Compared to active standing, rhythmic breathing increases LF (ms^2^) and LF (n.u.) for all groups and menstrual cycle phases, and increases HF (ms^2^), HF (n.u.) and (LF/HF) for all healthy women and for women with T2DM only during postmenopause.

### Associations between sex hormones and HRV indices

As an initial approach to describe the presence of relations between HRV and sex hormones, bivariate Pearson correlation coefficients between HRV indices, sex hormones, age, and glucose were calculated, using each measurement as an independent variable and we gathered the complete and combined sample of healthy women and women with T2DM during premenopause (including each of the three phases of the menstrual cycle: proliferative, luteal and menses) and postmenopausal groups (n=118). We also run bivariate Pearson correlation coefficients for each category as premenopause or postmenopause and in each phase of the menstrual cycle, but the correlations were maintained.

Table 3 shows the bivariate Pearson correlation coefficients between HRV indices, sex hormones, age, and glucose. MeanNN does not significantly correlate with sex hormones in any physiological condition but has weak negative correlations with age and glucose in the supine position. SDNN, RMSSD, pNN20, LF (ms^2^), and HF (ms^2^) have positive correlations with estradiol, progesterone and testosterone, and negative correlations with LH and FSH, during the supine position, and most of these correlations remain significant during the physiological conditions of active standing and rhythmic breathing. In general, correlations with sex hormones are weak, except for estradiol where correlation strength becomes moderate. Additionally, SDNN, RMSSD, pNN20, LF (ms^2^), and HF (ms^2^) have weak to moderate negative correlations with age in all conditions and weak correlations with glucose during supine position and rhythmic breathing. Meanwhile, LF (n.u.), HF (n.u.), and LF/HF do not correlate with sex hormones in any physiological conditions, except for testosterone which shows weak correlations during active standing. These HRV indices are not correlated with age or glucose in any condition, except for glucose which weakly correlates in the supine position. Finally, all HRV indices correlate with MeanNN in all conditions (weak to moderate correlations), except for LF (ms^2^) during active standing, as well as LF (n.u.), HF (n.u) and LF/HF during rhythmic breathing.

**Table 3:** Bivariate Pearson correlations between HRV indices, sex hormone levels, age and glucose, calculated using the complete and combined sample of healthy women and women with T2DM during premenopause (including each of the three phases of the menstrual cycle) and postmenopausal and premenopausal groups (n=118).

	LogEstradiol	LogProgesterone	Testosterone	LogLH	LogFSH	Age	Glucose	MeanNN
**Supine position**								
MeanNN	0.080	0.078	0.122	−0.093	−0.059	−0.244**	−0.234*	---
LogSDNN	0.368**	0.258**	0.321**	−0.191*	−0.303**	−0.431**	−0.107	0.512**
LogRMSSD	0.404**	0.300**	0.243**	−0.258**	−0.346**	−0.526**	−0.324**	0.682**
pNN20	0.387**	0.236*	0.190*	−0.301**	−0.351**	−0.530**	−0.298**	0.660**
LogPLF	0.375**	0.204*	0.391**	−0.138	−0.261**	−0.437**	−0.012	0.347**
LogPHF	0.414**	0.296**	0.194*	−0.233*	−0.349**	−0.489**	−0.354**	0.553**
LFNorm	−0.093	−0.145	0.156	0.151	0.149	0.142	0.365**	−0.282**
HFNorm	0.099	0.151	−0.157	−0.154	−0.154	−0.143	−0.365**	0.277**
LogLFHF	−0.087	−0.126	0.183*	0.122	0.127	0.106	0.391**	−0.270**
Active standing								
MeanNN	−0.106	−0.108	−0.058	0.086	0.166	0.155	−0.024	-
LogSDNN	0.392**	0.231*	0.266**	−0.142	−0.251**	−0.381**	−0.048	0.336**
LogRMSSD	0.373**	0.199*	0.146	−0.107	−0.193*	−0.256**	−0.088	0.568**
pNN20	0.322**	0.183*	0.081	−0.133	−0.194*	−0.231*	−0.036	0.617**
LogPLF	0.466**	0.269**	0.339**	−0.126	−0.295**	−0.446**	0.022	0.109
LogPHF	0.402**	0.247**	0.138	−0.129	−0.238**	−0.300**	−0.103	0.392**
LFNorm	0.007	−0.006	0.203*	0.049	−0.013	−0.085	0.150	−0.334**
HFNorm	−0.011	−0.009	−0.200*	−0.051	0.014	0.084	−0.139	0.327**
LogLFHF	0.032	−0.002	0.203*	0.017	−0.037	−0.126	0.150	−0.348**
Rhythmic breathing								
MeanNN	0.021	−0.011	0.082	−0.033	0.005	−0.137	−0.143	−−−
LogSDNN	0.273**	0.306**	0.222*	−0.305**	−0.291**	−0.448**	−0.287**	0.436**
LogRMSSD	0.249**	0.250**	0.145	−0.257**	−0.233*	−0.404**	−0.297**	0.547**
pNN20	0.159	0.139	0.089	−0.131	−0.182	−0.267**	−0.152	0.496**
LogPLF	0.266**	0.326**	0.171	−0.2()93**	−0.296**	−0.441**	−0.297**	0.409**
LogPHF	0.237*	0.251**	0.107	−0.239**	−0.221*	−0.370**	−0.227*	0.342**
LFNorm	0.006	0.033	0.099	−0.056	−0.095	−0.030	0.011	0.041
HFNorm	−0.005	−0.028	−0.101	0.053	0.093	0.027	−0.013	−0.036
LogLFHF	−0.010	0.052	0.070	−0.024	−0.060	−0.017	−0.045	0.018

* p < 0.05, ** p < 0.01

Significant bivariate correlations were further explored through multiple linear regression analysis. As an example, Table 4 displays the multiple linear regression models for HRV indices, MeanNN, and estradiol. MeanNN was included as an independent variable in these regression models due to the strong dependence of the HRV indices on MeanNN [20,40]. In most conditions, both MeanNN and estradiol exhibit significant independent associations with all HRV indices.

**Table 4:** Example of a multiple linear regression model for HRV indices (dependent variables) and MeanNN and estradiol (independent variables). HRV indices and MeanNN were measured in the supine position, active standing, and rhythmic breathing. All regression models included the complete and combined sample of healthy women and women with T2DM during premenopause (mean over the three phases of the menstrual cycle) and postmenopause (n=69).

Variables	Standardized β^^^	β^^^	CI95%		P-value	Adjusted R^2^
**Predicted HRV index: Log(SDNN) (s) in supine position**						
*Intercept*		−2.321	−2.640	−2.002	<0.001	0.427
*MeanNN(s)*	0.470	0.766	0.437	1.096	<0.001	
*LogEstradiol(pg/ml)*	0.401	0.143	0.071	0.215	<0.001	
**Predicted HRV index: Log(RMSSD) (s) in supine position**						
*Intercept*		0.043	−0.296	0.383	0.800	0.598
*MeanNN(s)*	0.608	1.261	0.910	1.612	<0.001	
*LogEstradiol(pg/ml)*	0.397	0.180	0.103	0.257	<0.001	
**Predicted HRV index: pNN20 (%) in supine position**						
*Intercept*		−32.592	−46.761	−18.423	<0.001	0.581
*MeanNN(s)*	0.609	51.596	36.947	66.244	<0.001	
*LogEstradiol(pg/ml)*	0.379	7.012	3.810	10.213	<0.001	
**Predicted HRV index: Log(LF) (ms** ^**2**^ **) in supine position**						
*Intercept*		−5.082	−5.815	−4.349	<0.001	0.352
*MeanNN(s)*	0.340	1.200	0.442	1.958	0.002	
*LogEstradiol(pg/ml)*	0.453	0.349	0.183	0.515	<0.001	
**Predicted HRV index: Log(HF)(ms** ^**2**^ **) in supine position**						
*Intercept*		−6.038	−6.760	−5.316	<0.001	0.497
*MeanNN(s)*	0.507	1.997	1.250	2.744	<0.001	
*LogEstradiol(pg/ml)*	0.427	0.368	0.205	0.531	<0.001	
**Predicted HRV index: Log(SDNN)(s) in active standing**						
*Intercept*		−2.186	−2.516	−1.855	<0.001	0.312
*MeanNN(s)*	0.412	0.700	0.327	1.073	<0.001	
*LogEstradiol(pg/ml)*	0.455	0.136	0.070	0.202	<0.001	
**Predicted HRV index: Log(RMSSD)(s) in active standing**						
*Intercept*		0.022	−0.330	0.375	0.901	0.504
*MeanNN(s)*	0.580	1.239	0.840	1.637	<0.001	
*LogEstradiol(pg/ml)*	0.500	0.188	0.118	0.258	<0.001	
**Predicted HRV index: pNN20(%) in active standing**						
*Intercept*		−45.828	−63.350	−28.281	<0.001	0.478
*MeanNN(s)*	0.621	64.315	44.497	84.133	<0.001	
*LogEstradiol(pg/ml)*	0.408	7.435	3.951	10.919	<0.001	
**Predicted HRV index: Log(LF)(ms** ^**2**^ **) in active standing**						
*Intercept*		−4.764	−5.578	−3.950	<0.001	0.307
*MeanNN(s)*	0.221	0.922	0.003	1.842	0.049	
*LogEstradiol(pg/ml)*	0.557	0.409	0.247	0.570	<0.001	
**Predicted HRV index: Log(HF)(ms** ^**2**^ **) in active standing**						
*Intercept*		−6.205	−7.063	−5.346	<0.001	0.382
*MeanNN(s)*	0.438	2.043	1.073	3.012	<0.001	
*LogEstradiol(pg/ml)*	0.511	0.419	0.248	0.589	<0.001	
**Predicted HRV index: Log(SDNN)(s) in rhythmic breathing**						
*Intercept*		−1.798	−2.117	−1.479	<0.001	0.258
*MeanNN(s)*	0.416	0.567	0.257	0.878	0.001	
*LogEstradiol(pg/ml)*	0.299	0.092	0.022	0.162	0.011	
**Predicted HRV index: Log(RMSSD)(s) in rhythmic breathing**						
*Intercept*		0.674	0.284	1.065	0.001	0.339
*MeanNN(s)*	0.531	0.940	0.560	1.321	<0.001	
*LogEstradiol(pg/ml)*	0.241	0.096	0.011	0.182	0.028	
**Predicted HRV index: pNN20(%) in rhythmic breathing**						
*Intercept*		2.801	−10.032	15.634	0.664	0.203
*MeanNN(s)*	0.426	22.543	10.056	35.030	0.001	
*LogEstradiol(pg/ml)*	0.189	2.248	−0.557	5.053	0.114	
**Predicted HRV index: Log(LF)(ms** ^**2**^ **) in rhythmic breathing**						
*Intercept*		−3.818	−4.521	−3.114	<0.001	0.230
*MeanNN(s)*	0.378	1.117	0.432	1.802	0.002	
*LogEstradiol(pg/ml)*	0.306	0.203	0.049	0.357	0.011	
**Predicted HRV index: Log(HF)(ms** ^**2**^ **) in rhythmic breathing**						
*Intercept*		−4.965	−5.867	−4.062	<0.001	0.176
*MeanNN(s)*	0.369	1.354	0.476	2.232	0.003	
*LogEstradiol(pg/ml)*	0.231	0.190	−0.007	0.387	0.059	

Table 5 summarizes the multiple linear regression analyses for all HRV indices (as dependent variables), considering sex hormones and MeanNN as independent variables. Overall, after adjusting for MeanNN, all sex hormones maintain an independent association with HRV indices in most conditions.

**Table 5:** Summary of significant p-values (p<0.05) from specific linear multiple regression models adjusted for MeanNN and sex hormones. Each HRV index was modeled with each sex hormone as an independent variable during the three maneuvers. All regression models included the complete and combined sample of healthy women and women with T2DM during premenopause (mean over the three phases of the menstrual cycle) and postmenopause (n = 69).

	Supine position					Active standing					Rhythmic breathing				
	SDNN	RMSSD	pNN20	LF	HF	SDNN	RMSSD	pNN20	LF	HF	SDNN	RMSSD	pNN20	LF	HF
**LogEstradiol**	✓	✓	✓	✓	✓	✓	✓	✓	✓	✓	✓	✓	X	X	X
**Testosterone**	✓	✓	X	✓	X	✓	✓	X	✓	✓	✓	X	X	X	X
**LogProgesterone**	✓	✓	✓	✓	✓	✓	✓	✓	✓	✓	✓	✓	✓	✓	✓
**LogLH**	X	✓	✓	X	✓	✓	X	✓	X	✓	✓	✓	X	✓	✓
**LogFSH**	✓	✓	✓	✓	✓	✓	✓	✓	✓	✓	✓	✓	X	✓	✓

“✓”sex hormones were statistically significant predictors.

“X” sex hormones were not statistically significant predictors.

Table 6 shows linear regression analyses where age and glucose are included as additional independent variables, with estradiol maintaining a significant independent association.

**Table 6:** Multiple linear regression models adjusted for MeanNN, estradiol, age, and glucose level. The models include the complete and combined sample of healthy women and women with T2DM during premenopause (mean over the three phases of the menstrual cycle) and postmenopause (n=69).

Variables	Standardized <<Eqn3>>	<<Eqn4>>	CI95%		p-value	Adjusted R^2^
**Predicted HRV index: Log (SDNN) in supine position**						
* Intercept*		−2.208	−2.818	−1.598	<0.001	0.412
* MeanNN(s)*	0.467	0.761	0.424	1.099	<0.001	
* LogEstradiol(pg/ml)*	0.327	0.117	0.001	0.233	0.049	
* Age (years)*	−0.114	−0.003	−0.010	0.005	0.503	
* Glucose (mg/dL)*	0.052	0.001	−0.002	0.003	0.632	
**Predicted HRV index: LogRMSSD in active standing**						
* Intercept*		0.221	−0.325	0.767	0.421	0.495
* MeanNN(s)*	0.594	1.268	0.861	1.676	<0.001	
* LogEstradiol(pg/ml)*	0.391	0.147	0.033	0.260	0.012	
* Age (years)*	−0.132	−0.003	−0.011	0.004	0.404	
* Glucose (mg/dL)*	−0.024	<0.001	−0.003	0.002	0.809	
**Predicted HRV index: pNN20 in active standing**						
* Intercept*		−39.515	−66.718	−12.313	0.005	0.467
* MeanNN(s)*	0.631	65.349	45.078	85.621	<0.001	
* LogEstradiol(pg/ml)*	0.315	5.737	0.091	11.382	0.047	
* Age (years)*	−0.142	−0.161	−0.530	0.208	0.384	
* Glucose (mg/dL)*	0.060	0.033	−0.080	0.145	0.562	

Table 7 summarizes the multiple linear regression analyses for all HRV indices (as dependent variables), considering the sex hormones, MeanNN, age and glucose as independent variables. Overall, after adjusting for MeanNN, regression models confirm a significant association between all sex hormones and HRV indices. Estradiol maintains a significant correlation even after controlling also for age and glucose.

**Table 7:** Summary of significant p-values (p<0.05) from specific linear multiple regression models adjusted by MeanNN, sex hormones, age and glucose level. Each sex hormone was established as an independent variable for each HRV index in the three maneuvers. All regression models included the complete and combined sample of healthy women and women withT2DM during premenopause (mean over the three phases of the menstrual cycle) and postmenopause (n = 69).

	Supine position					Active standing					Rhythmic breathing				
	SDNN	RMSSD	pNN20	PLF	PHF	SDNN	RMSSD	pNN20	PLF	PHF	SDNN	RMSSD	pNN20	PLF	PHF
**LogEstradiol**	✓	X	X	X	X	X	✓	✓	X	X	X	X	X	X	X
**Testosterona**	X	X	X	X	X	X	X	X	X	X	X	X	X	X	X
**LogProgesterona**	X	X	X	X	X	X	X	X	X	X	X	X	X	X	X
**LogLH**	X	X	X	X	X	X	X	X	X	X	X	X	X	X	X
**LogFSH**	X	X	X	X	X	X	X	X	X	X	X	X	X	X	X

“✓”sex hormones were statistically significant predictors.

“X” sex hormones were not statistically s

## 4. Discussion

### Main contribution

This study is the first to describe the relationship between sex hormones and HRV indices in women with well-controlled T2DM and healthy women. In addition, it provides comparisons of different phases of the menstrual cycle (including postmenopause) and different physiological conditions (supine, standing and rhythmic breathing). The results demonstrate correlations between all sex hormones and HRV indices that are independent of MeanNN but not of age or glucose level, except for estradiol in three time-domain indices.

### Comparisons of sex hormones and HRV

In the group comparisons, there are no significant differences in sex hormone levels between healthy women and women with T2DM, suggesting that endocrine regulation is preserved in women with well-controlled T2DM. Some studies that included both men and women found differences in testosterone and estrogen levels between healthy controls and patients with T2DM [41,42], particularly in postmenopausal women with T2DM and age-matched men with T2DM [43], which is contrary to the present results. These differences in sex hormone levels may be due that the women with T2DM in this study were all well-controlled, while the literature includes diabetic patients with a variety of health conditions.

In the present sample, HRV indices are similar between healthy women and women with T2DM, indicating that autonomic regulation is conserved in women with well-controlled T2DM, as reported previously [20]. Other research articles have compared individuals with early or late diabetes diagnosis, as well as short- and long-standing diabetes with a heterogenous glycemic control. These studies reported decreased HRV, particularly in those with long-standing diabetes and poor glycemic control [19].

### Relations between HRV and sex hormones

Few studies have addressed the relationship between sex hormones and HRV indices in both healthy individuals and patients with T2DM. Some research articles have only considered differences between genders [44–46], while other studies have compared different phases of the menstrual cycle without measuring sex hormone levels [47–50].

In healthy women, there is evidence supporting a positive relation between sex hormones and time-frequency HRV [24]. Moreover, the effect of hormone therapy (combined and estradiol only) on HRV indices was reported in postmenopausal healthy women [32,33]. In contrast, no association between estradiol and HRV indices has been reported in premenopausal women during different phases of the menstrual cycle [34] or in menopausal women with or without hormone therapy [26,34]. Furthermore, there are no studies in T2DM. Only one study in adolescent women (13–16 years old) with type 1 diabetes showed correlations between HRV indices and androgens (free androgen index FAI), sex hormone binding protein (SHBP), glucose levels, and insulin [28].

In the present study with healthy women and women with T2DM, significant bivariate correlations exist between all sex hormones and HRV indices. These correlations persist under three physiological conditions: supine, active standing, and rhythmic breathing, suggesting that the mechanisms that could associate both systems are present even with physiological challenges. The hypothesis tested was correct in the direction of some of the correlations but not in all the indices nor all the hormones evaluated. The correlation is positive with estradiol, testosterone and progesterone, and negative with LH and FSH. A possible explanation is that estradiol and progesterone induce higher parasympathetic activity via autonomic centers in the nervous system [51,52], whereas, testosterone increases the cardiomotor vagal activity [53].

In the present contribution, 17β-estradiol, the most abundant estrogen in women, maintained a significant independent contribution to HRV indices. Estrogens may influence HRV through several mechanisms. Some authors suggest that estrogens increase cardiac vagal modulation [32,54]. Estrogens increase the activity of choline acetyltransferase, the production of acetylcholine and choline uptake in cardiac autonomic nerves [55]. On the other hand, estrogen also inhibits the activity of the enzyme tyrosine hydroxylase involved in catecholamines synthesis and enhances the presynaptic inhibitory pathway to release noradrenaline (α2-adrenoceptors) in synaptic ends and from the adrenal glands.

Moreover, in the central nervous system, estrogens can modulate autonomic tone using both receptors alpha (ERα) and beta (Erβ) located in different nuclei, such as the ambiguous nucleus (increasing activity and the vagal tone), and the rostral ventrolateral medulla (decreasing sympathetic tone). Therefore, estrogens increase the levels and effect of gamma aminobutyric acid (GABA) and decrease glutamate levels in some autonomic neurons of the parabrachial nucleus, decreasing its activity and the sympathetic response [56].

The multiple regression analyses show that the association between HRV indices and sex hormones is independent from MeanNN. Estradiol maintains significant associations with HRV indices even after controlling also for age and glucose levels. Aging has been related to changes in the autonomic balance, increasing sympathetic and decreasing parasympathetic activity [57], while glycemia, causes changes in HRV indices in metabolic syndrome, type 1 [28] and type 2 diabetes mellitus. Similar to the current results, in studies on type 1 diabetes found that the association between androgens and HRV was not independent of glycemia, weight, or SHBP, supporting the importance of glycemia as an independent variable in predicting HRV indices [28].

An important finding is that in our study the associations between HRV indices and sex hormones remain significant even when MeanNN is added to the models. Given the strong relation between HRV and MeanNN [58], some authors have contested the capacity of HRV as an independent measure of ANS activity beyond what may be derived from the heart rate (HR) [40,59]. However, evidence has recently been published that the relationship between HRV and MeanNN depends on a variety of internal and external factors and may therefore serve to characterize the underlying health state and in particular T2DM [20]. The evidence from the current study supports the hypothesis that other independent factors than MeanNN can predict HRV indices. In this case, sex hormones are independent from MeanNN (i.e., without significant bivariate correlations). It is emphasized that HRV studies should consider MeanNN and sex hormones as part of the independent variables that influence HRV indices.

### Physiological and clinical implications

In this study, we observed that estradiol levels modulate HRV indices and should be considered in cardiovascular and autonomic homeostasis. We propose that the failure of both regulatory systems could synergize a pathological process causing CVDs in women with T2DM. The relationship between both systems should be described and elucidated if we expect to use these as a biomarker to establish the borders between cardiovascular health and disease.

Contrary to what was expected, no significant differences were found between the control group and the T2DM group. This result may be due to the clinical control of the T2DM group, as this group had glucose levels almost within normal values, without comorbidities, and most of them were taking only metformin as the normoglycemic treatment. This result is a strong defender that the maintenance of glycemia is the most important factor avoiding diabetic comorbidities.

Insulin resistance and sensitivity were not measured, since these women were diagnosed with T2DM previous to the study, and there is evidence that alterations in HRV are mediated by hyperglycemia rather than insulin [60,61].

Another important finding was that changes in the MeanNN and HRV indices did not differ between the healthy and T2DM groups when considering the mean values in each maneuver. However, significant differences were found in the response to autonomic challenges, such as active standing or rhythmic breathing with respect to the supine position, between the healthy and T2DM groups. This discovery suggests that when different maneuvers are applied, the magnitude of response reflects regulatory capacity and may serve to determine alterations.

There is clinical and epidemiological evidence that cardiovascular risk is disproportionally increased in postmenopausal women with diabetes [9–11]. HRV is widely used as a non-invasive proxy for assessing cardiovascular risk [62]. The present findings indicate that age, sex hormones and glucose are variables that influence HRV and may also be risk factors that contribute to the development of cardiovascular disease. This offers a possible explanation for the increased prevalence of cardiovascular disease in postmenopausal women with diabetes, where several of these risk factors are combined. Consequently, it is proposed that sex hormones, especially estrogen, should be considered in HRV research in the field of cardiovascular and autonomic sciences.

### Limitations of the study

The results presented in this contribution may not be representative for the whole population since the population sample comprises well-controlled diabetic patients under strict inclusion criteria to increase the internal validity of the study, which may limit generalizability. Further studies are needed to include different samples of patients with T2DM patients.

Age is the most important confounding factor in this study. The age of the participants cannot be controlled to reduce the age gap between premenopausal and postmenopausal women since aging is related to the physiology of these life stages.

Other independent variables, such as T2DM duration, diet, exercise, socioeconomic status, insulin resistance indices or educational factors, were not considered in this manuscript.

Each group had a different number of participants, with repeated measures only for the premenopausal group. The study design included three measures in premenopause and one in postmenopause, so we decided to average the premenopausal measurements to cancel out the repeated measures.. In the analysis of variance, the main effect of T2DM was non-significant for all sex hormones and HRV indices. However, due to the small sample size of some subgroups, the power to detect differences between the T2DM and healthy groups was low. Nevertheless, the primary question regarding the relationship between sex hormones and HRV indices was adequately addressed with a sufficient sample size and sufficient statistical power (see S1 File).

The timing of menstrual cycle phases was estimated based on self-reported regular cycles and the first day of menstrual bleeding, and ovulation was inferred but not experimentally confirmed. It is possible that the exact date of each phase did not correspond to the estimated dates.

## 5. Conclusions

The goal of this paper was to evaluate the correlation between sex hormones and heart rate variability (HRV) indices, in healthy and well-controlled type 2 diabetes women. Bivariate Pearson correlations showed that all sex hormones correlate with all HRV indices. In multiple linear regression models, estradiol is an independent predictor for HRV indices in supine position and active standing when including age, glucose level and MeanNN as covariables, indicating the interrelations between two of the major regulatory systems of the human body, i.e., the endocrine system and the autonomous nervous system.

In the intergroup analysis, it was shown that T2DM had no effect on the production of sex hormones or on the majority of the HRV indices in the studied population. This suggests that good glycemic control helps to preserve cardiac autonomic regulation as assessed through HRV.

All sex hormone levels correlate with HRV indices, and these relations are independent of MeanNN, but not independent of age and glucose level, except for estradiol. Estradiol is a predictor of SDNN in the supine position, RMSSD, and pNN20 in active standing, in all adjusted models. These findings suggest that women with well-controlled T2DM have preserved endocrine, cardiovascular, and autonomic nervous system functions, and that estradiol is the sex hormone related to autonomic and cardiovascular physiology and should be considered in research in women with different health status such as T2DM.

## Supporting information

S1 File Example of calculation of estimation of effect size and achieved statistical power in ANOVA of sex hormones in the inter-subject effect test. (DOCX)

S2 File Data. (XLSX)
