# Supplementary material for: Sex hormones correlate with heart rate variability in healthy women and this correlation is conserved in women with well-controlled type 2 diabetes mellitus
Source: PLoS One. 2025 Apr 23;20(4):e0320982. doi: 10.1371/journal.pone.0320982 (PMC12017505; doi:10.1371/journal.pone.0320982)
Supplement: S1 File — (DOCX) [file pone.0320982.s001.docx]

**Supplementary Information**

Example of calculation of estimation of effect size and achieved statistical power in ANOVA of sex hormones in the inter-subject effect test.

|  | Total sample size=118 |  |  |  |
| --- | --- | --- | --- | --- |
|  | T2DM (yes: n = 29; no: n = 40) |  |  |  |
|  | Menstrual cycle phase |  |  |  |
|  | proliferative (n = 27) |  |  |  |
|  | luteal (n = 28) |  |  |  |
|  | menstrual (n = 25) |  |  |  |
|  | postmenopause (n = 38) |  |  |  |
|  |  |  |  |  |
|  |  |  |  |  |
| **ESTRADIOL** | Factors | p value | effect size | achieved power |
| Main effect | T2DM | 0.725 | 0.001 | 0.064 |
| Main effect | Menstrual cycle phase | <0.001 | 0.735 | 1.000 |
| Interaction term | T2DM*menstrual cycle phase | 0.918 | 0.004 | 0.080 |
|  |  |  |  |  |
| **PROGESTERONE** | Factors | p value | effect size | achieved power |
| Main effect | T2DM | 0.419 | 0.005 | 0.127 |
| Main effect | Menstrual cycle phase | <0.001 | 0.489 | 1.000 |
| Interaction term | T2DM*menstrual cycle phase | 0.967 | 0.002 | 0.065 |
|  |  |  |  |  |
| **TESTOSTERONE** | Factors | p value | effect size | achieved power |
| Main effect | T2DM | 0.589 | 0.002 | 0.084 |
| Main effect | Menstrual cycle phase | <0.001 | 0.180 | 0.994 |
| Interaction term | T2DM*menstrual cycle phase | 0.565 | 0.017 | 0.190 |
|  |  |  |  |  |
| **LH** | Factors | p value | effect size | achieved power |
| Main effect | T2DM | 0.665 | 0.002 | 0.071 |
| Main effect | Menstrual cycle phase | <0.001 | 0.487 | 1.000 |
| Interaction term | T2DM*menstrual cycle phase | 0.462 | 0.021 | 0.234 |
|  |  |  |  |  |
| **FSH** | Factors | p value | effect size | achieved power |
| Main effect | T2DM | 0.774 | 0.001 | 0.059 |
| Main effect | Menstrual cycle phase | <0.001 | 0.555 | 1.000 |
| Interaction term | T2DM*menstrual cycle phase | 0.899 | 0.005 | 0.086 |
